# Training of Psychiatry and Child Psychiatry Residents : Ethical Aspects

**DOI:** 10.1192/j.eurpsy.2025.1501

**Published:** 2025-08-26

**Authors:** S. Bourgou, R. Ghachem, S. Halayem, G. Amira

**Affiliations:** 1 National College of Psychiatry and Child Psychiatry, Tunis; 2 Child psychiatry Department, Mongi Slim Hospital, La Marsa, Tunisia

## Abstract

**Introduction:**

Ethical practices are fundamental to the training and professional development of psychiatry and child psychiatry residents. However, challenges such as stigmatization, discrimination, and ethical dilemmas in clinical and research settings can impact the quality of their training experience.

**Objectives:**

1. To assess the degree of exposure of residents to stigmatization during their residency training.
2. To explore the level of awareness among residents regarding the practice of ethics in their clinical and research activities.

**Methods:**

This is a cross-sectional study conducted by the National College of Psychiatry and Child Psychiatry from January 13 to January 16, 2024. An anonymous Google Forms questionnaire about ethic aspects during during residency training was sent to psychiatry and child psychiatry residents via the college email and private groups.

**Results:**

We received 71 responses. The participants had an average age of 29.9 years, with a sex ratio of 0.1. Among them, 50.7% were child psychiatry residents, and 49.3% were psychiatry residents. Residents reported experiencing discrimination in 49% of cases, with the following breakdown: from senior staff (61.1%), paramedical staff (47.2%), and doctors from other specialties (33.3%). The primary cause of discrimination was the residency level (56.3%). Residents reported experiencing discrimination in role distribution within the department (35.2%) and in scientific work (31%).

Regarding their thesis work, 8.5% of residents felt obliged in choosing their thesis supervisor, and 16.9% felt pressured in selecting their thesis topic. Residents submitted their thesis work to the local committee in 58.5% of cases, informed participants about the study in a satisfactory manner in 75.4% of cases, and obtained oral informed consent from participants (or their parents) in 46.2% of cases. The residents felt that training in psychiatric ethics was necessary in 94.5% of cases and that specific training in research ethics in psychiatry was necessary in 91.8% of cases.

**Conclusions:**

Taking into account the various findings of our survey and raising awareness among different stakeholders, including doctors and paramedical team members, are essential measures to ensure that psychiatry and child psychiatry residents can complete their specialty training while adhering to fundamental ethical principles.

**Disclosure of Interest:**

None Declared

